# Quantitative Trait Locus Analysis for Deep-Sowing Germination Ability in the Maize IBM Syn10 DH Population

**DOI:** 10.3389/fpls.2017.00813

**Published:** 2017-05-22

**Authors:** Hongjun Liu, Lin Zhang, Jiechen Wang, Changsheng Li, Xing Zeng, Shupeng Xie, Yongzhong Zhang, Sisi Liu, Songlin Hu, Jianhua Wang, Michael Lee, Thomas Lübberstedt, Guangwu Zhao

**Affiliations:** ^1^State Key Laboratory of Crop Biology, Shandong Key Laboratory of Crop Biology, College of Life Sciences, Shandong Agricultural UniversityTai'an, China; ^2^Department of Agronomy, Northeast Agricultural UniversityHarbin, China; ^3^National Key Laboratory of Plant Molecular Genetics, Institute of Plant Physiology & Ecology, Shanghai Institutes for Biological Sciences, Chinese Academy of SciencesShanghai, China; ^4^Department of Agronomy, Shenyang Agricultural UniversityShenyang, China; ^5^Suihua Sub-academy, Heilongjiang Academy of Agricultural SciencesSuihua, China; ^6^Department of Plant Genetics and Breeding, College of Agronomy Sciences, Shandong Agricultural UniversityTai'an, China; ^7^Key Laboratory of Biology and Genetic Improvement of Maize in Southwest Region, Maize Research Institute, Sichuan Agricultural UniversityChengdu, China; ^8^Department of Agronomy, Iowa State UniversityAmes, IA, United States; ^9^Department of Plant Genetics, Breeding and Seed Science, China Agricultural UniversityBeijing, China; ^10^The Key Laboratory for Quality Improvement of Agricultural Products of Zhejiang Province, College of Agriculture and Food Science, Zhejiang Agriculture and Forestry UniversityLin'an, China

**Keywords:** quantitative trait locus, deep-sowing, germination ability, maize, IBM Syn10 DH population

## Abstract

Deep-sowing is an effective measure to ensure seeds absorbing water from deep soil layer and emerging normally in arid and semiarid regions. However, existing varieties demonstrate poor germination ability in deep soil layer and some key quantitative trait loci (QTL) or genes related to deep-sowing germination ability remain to be identified and analyzed. In this study, a high-resolution genetic map based on 280 lines of the intermated B73 × Mo17 (IBM) Syn10 doubled haploid (DH) population which comprised 6618 bin markers was used for the QTL analysis of deep-sowing germination related traits. The results showed significant differences in germination related traits under deep-sowing condition (12.5 cm) and standard-germination condition (2 cm) between two parental lines. In total, 8, 11, 13, 15, and 18 QTL for germination rate, seedling length, mesocotyl length, plumule length, and coleoptile length were detected for the two sowing conditions, respectively. These QTL explained 2.51–7.8% of the phenotypic variance with LOD scores ranging from 2.52 to 7.13. Additionally, 32 overlapping QTL formed 11 QTL clusters on all chromosomes except for chromosome 8, indicating the minor effect genes have a pleiotropic role in regulating various traits. Furthermore, we identified six candidate genes related to deep-sowing germination ability, which were co-located in the cluster regions. The results provide a basis for molecular marker assisted breeding and functional study in deep-sowing germination ability of maize.

## Introduction

Maize (*Zea mays* L.) is the largest agricultural crop in the world based on acreage and yield (http://faostat.fao.org/). In China, maize has been cultivated on more than 34 million hectares since 2012. However, as two-third of the maize cultivation area is located in arid and semiarid regions, seedlings often experience severe drought stress (Iuchi et al., 2001; Tommasini et al., 2008; Christov et al., 2014). To overcome this issue, deep-sowing strategy, an effective measure to ensure that seeds absorb water from deeper soil layers to germinate normally, has been applied (Zhao et al., 2010). However, existing varieties show poor germination ability when sown into deeper soil layers (Zhao and Wang, 2008). Therefore, it is critical to understand and explore how key quantitative trait loci (QTL) or genes are related to deep-sowing germination ability for more efficient breeding of varieties adapted to these (semi-) arid regions.

Although several QTL for deep-sowing tolerance-related traits in maize have been identified (Zhang et al., 2012), questions regarding their effects remain unresolved owing to the utilization of low density of molecular markers and limited population sizes. Recent advances in next-generation sequencing technologies provide an effective platform for detection of high-quality single nucleotide polymorphism (SNP) markers for genotyping of mapping populations (Varshney et al., 2009; Wang et al., 2015; Raihan et al., 2016). Genotype-by-sequencing (GBS) is a popular new method for affordably acquiring dense genome wide marker data for large populations and has been successfully utilized for highly diverse species (Elshire et al., 2011). However, GBS presents limitations with a relatively large proportion of missing data and a small, but rarely corrected, percentage of SNP genotyping sequencing errors (Chen H. et al., 2014; Chen W. et al., 2014). A good option for imputing missing SNP data is the sliding-window approach, where adjacent SNPs with same genotype in an interval are combined into bins that demarcate recombination locations across the whole population (Huang et al., 2009). This method is more powerful for detecting QTL than traditional methods and has also been employed for fine mapping of some important agronomic traits in rice and maize (Gao et al., 2013; Chen Z. et al., 2014; Liu et al., 2015, 2016).

In maize, improved genetic resolution could be achieved very easily by randomly intermating plants in the F2 generation prior to the derivation of mapping progeny (Lee et al., 2002). The IBM Syn10 doubled haploid population (IBM Syn10 DH) was obtained after 10 generations of intermating and then followed by DH line development (Hussain et al., 2007). Compared to F_2_-derived RILs, the IBM Syn10 DH population exhibits a higher genetic resolution than the earlier Syn4 RIL population, with almost two-fold increase in the genetic map length and is therefore more suitable for high density genetic linkage map construction, QTL fine mapping of important traits, positional cloning and functional studies of target genes (Holloway et al., 2011; Liu et al., 2015).

Traits, such as germination rate, mesocotyl length, coleoptile length, plumule length, and seedling length are affected by sowing depths. In this study, we measured them on individual plants and used for QTL mapping. Under the conditions of deep-sowing, mesocotyl, coleoptile, and plumule elongation are vital for seed germination from the deep layer of soil. The objectives of this study were: (i) identifying QTL controlling deep-sowing germination ability in the maize IBM Syn10 population; (ii) exploring QTL that are co-localized in the same regions between different phenotypic traits under two sowing conditions; and (iii) mining candidate genes related to deep-sowing germination ability. The results of this study provide a basis for further fine mapping, molecular marker assisted breeding and functional study in deep-sowing germination ability of maize.

## Materials and methods

### Plant materials

Seeds of 280 lines of IBM Syn10 DH population and their parents B73 and Mo17 were produced in summer 2010 by manual self-pollination of each plant at Agronomy Farm of Iowa State University (ISU), Ames, Iowa, USA, located at 42°02′05″N, 93°37′12″W. This region of Iowa has a humid continental climate with 865.4 mm of average annual precipitation. After maturation, seeds were harvested and dried for 7 days at ambient temperature and forced air at the Agronomy Research Center, ISU. Seeds were subsequently stored at 4°C and 50% relative humidity before use in seed storage facility of Agronomy Hall, ISU. The average standard germination rate of B73, Mo17, and IBM Syn10 population was 100.0, 78.0, and 73.1%, respectively.

### Phenotypic evaluation of germination ability and statistical data analysis

Germination experiments were performed using sand-beds in a greenhouse, Department of Agronomy, ISU. Sand was first sterilized at 180°C for 1 h and passed through a 2.0 mm diameter sieve before watering to 60% moisture content. Sand was then poured on a greenhouse bench at 23.5 cm depth. Next, seeds were placed on the surface of the sand bench and then covered with 12.5 cm (In our preliminary experiment, 12.5 cm is more suitable sowing depth to differentiate germination ability than 10 or 15 cm, data not shown) or 2 cm deep layer of sand. To prevent loss of moisture and to keep the seeds in dark, the sand benches were covered with two layers of thick black plastic bags. Fifteen seeds per line and per replicate were incubated at 28°C. The experiments were conducted in a randomized complete block design with three replicates for each line. So, total 45 seeds were used for each line. After 7 days incubation, germination rate per experimental unit was determined according to the rate of seedlings that reached or exceeded 1 cm above the surface of sand. Then, seed and seedling were taken out from the sand and the lengths of mesocotyl, coleoptile, plumule, and seedling were measured using a straight ruler. Under the conditions of deep-sowing (12.5 cm) and standard-germination (2 cm), germination rate, mesocotyl length, coleoptile length, plumule length, seedling length were abbreviated as DSGR, DSML, DSCL, DSPL, DSSL and SGGR, SGML, SGCL, SGPL, SGSL, respectively.

Significance tests for all traits between the two parental lines B73 and Mo17 were conducted using Student's *t*-test. The data of 280 IBM lines were analyzed using one-way analysis of variance (ANOVA), which was carried out with the PROC GLM procedure in SAS 8.2 (SAS, Inc., Cary, NC, USA). Repeatability for each trait is here indicated as *R*^*2*^ (%) and it was calculated based on an ANOVA as *R*^*2*^ = [($S_{t}^{\text{2}}$- $S_{e}^{\text{2}}$)/r]/[($S_{t}^{2}$- $S_{e}^{\text{2}}$)/r+$S_{e}^{\text{2}}$] × 100. Where *R*^*2*^, $S_{t}^{\text{2}}$, $S_{e}^{\text{2}}$, *r* indicates repeatability, treatment variance, error variance, the number of replicates, respectively. The coefficients of variation (CV, %) for each trait were calculated as follows: CV = s /$\bar{x}$, where s is the standard deviation (Hu et al., 2016).

### Genotyping and QTL analysis

IBM genotypic data were downloaded from CyVerse (http://www.cyverse.org/discovery-environment). First, we re-sequenced the inbred line Mo17 with a 26.5x depth to construct a custom-made Mo17 pseudomolecule SNP reference map, and calling the SNPs between Mo17 and B73. Subsequently, the reads and SNPs for each IBM Syn10 DH line were compared with Mo17 and B73 reference SNPs. By using a sliding window approach (15 SNPs), we constructed a bin map with 6,618 recombination bins for QTL analysis (Liu et al., 2015). Genetic mapping was performed by standard procedures (Van Ooijen, 2006; Wu et al., 2008). QTL analysis was performed using the high-resolution genetic map by QTL Cartographer Unix version 1.17f (Wang and Zeng, 2005), We selected 2.5 as the logarithm of odds (LOD) threshold values for each trait. Composite Interval Mapping (CIM) pattern was used for QTL analysis (Li et al., 2008).

### Candidate-gene selection, RNA extraction, and quantification

Totally, 107 genes that have annotations were extracted from five overlapping intervals based on B73_RefGen_v3 in Maize Genetics and Genomics Database (http://www.maizegdb.org) (Supplementary Table 1). Combined with the results of our previous study (Zhao et al., 2010), six candidate genes that are related to deep-sowing germination traits were selected for real-time PCR validation from two overlapping regions that only associate to deep-sowing germination traits (*qDSPL4-1* and *qDSSL4-1* overlapping region between 82.25 and 127.075 Mb on chromosome 4, *qDSML7-1* and *qDSSL7-1* overlapping region between 125.55 and 129.8 Mb on chromosome 7). To deeply analyze their differential expressions between B73 and Mo17, total RNA of the 7-day-old seedlings of the parental lines at 12.5 cm sowing depth in darkness was extracted with TRIzol (Invitrogen, USA) and purified with the RNeasy Mini Kit after digestion with DNase1 (Qiagen, Germany). Two micrograms of RNA in a 20 μl reaction system was employed for cDNA synthesis with the SuperScript III First Strand Kit (Invitrogen). qPCR was performed using SYBR® Green (Takara) with the Bio-Rad CFX Connect by using the *Actin* gene as control, and the comparative CT method (ΔΔCT method) was employed to calculate relative gene expression. In qPCR reaction system, cDNA was firstly pre-denaturated at 95°C for 30 s, and the condition of 40 cycles of PCR amplified reaction was then set to 95°C denaturation for 5 s, 60°C annealing for 34 s, 72°C extension for 30 s. All the expression data used for analysis had at least five biological samples, each with three technical replications. Student's *t*-test was used to determine statistical significance. All primers were listed in Supplementary Table 2.

## Results

### Phenotypic analysis of germination-related traits at different sowing depths

**Figure 1:**
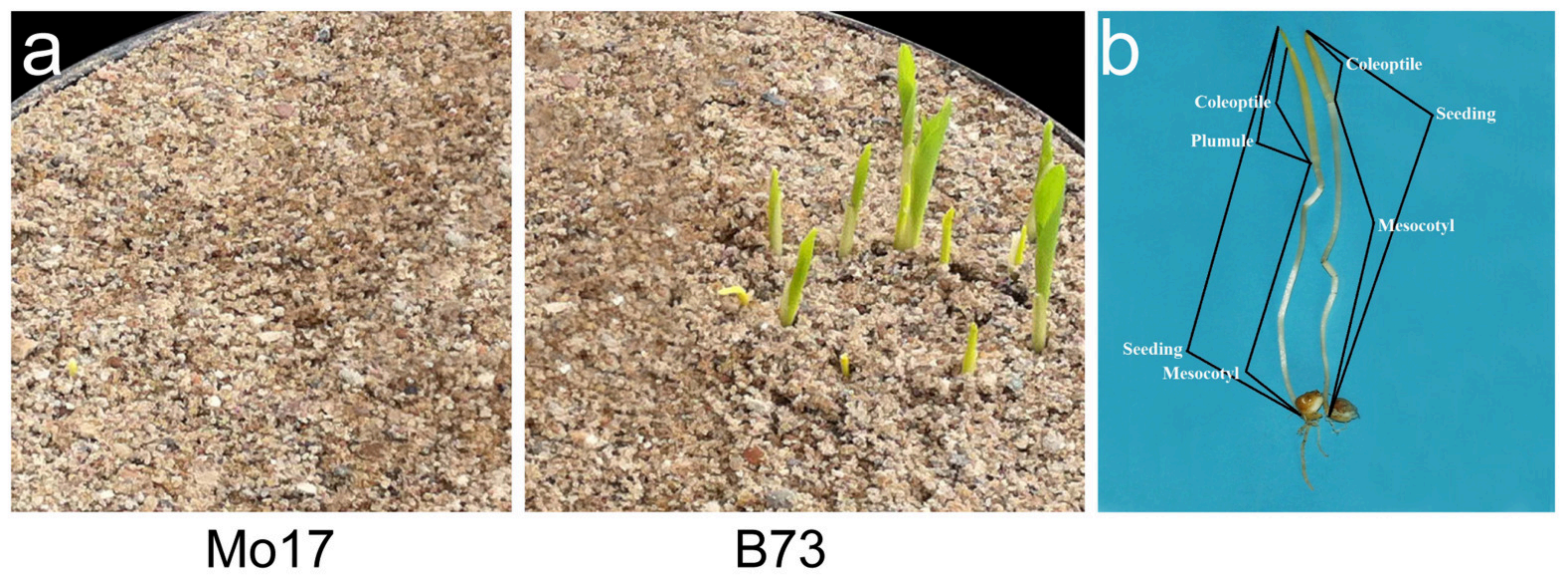
**Morphological observation of germinated seeds at 7 days under 12.5 cm sowing depth. (a)** Deep-sowing germination of Mo17 and B73; **(b)** The structure of seedlings.

**Figure 2:**
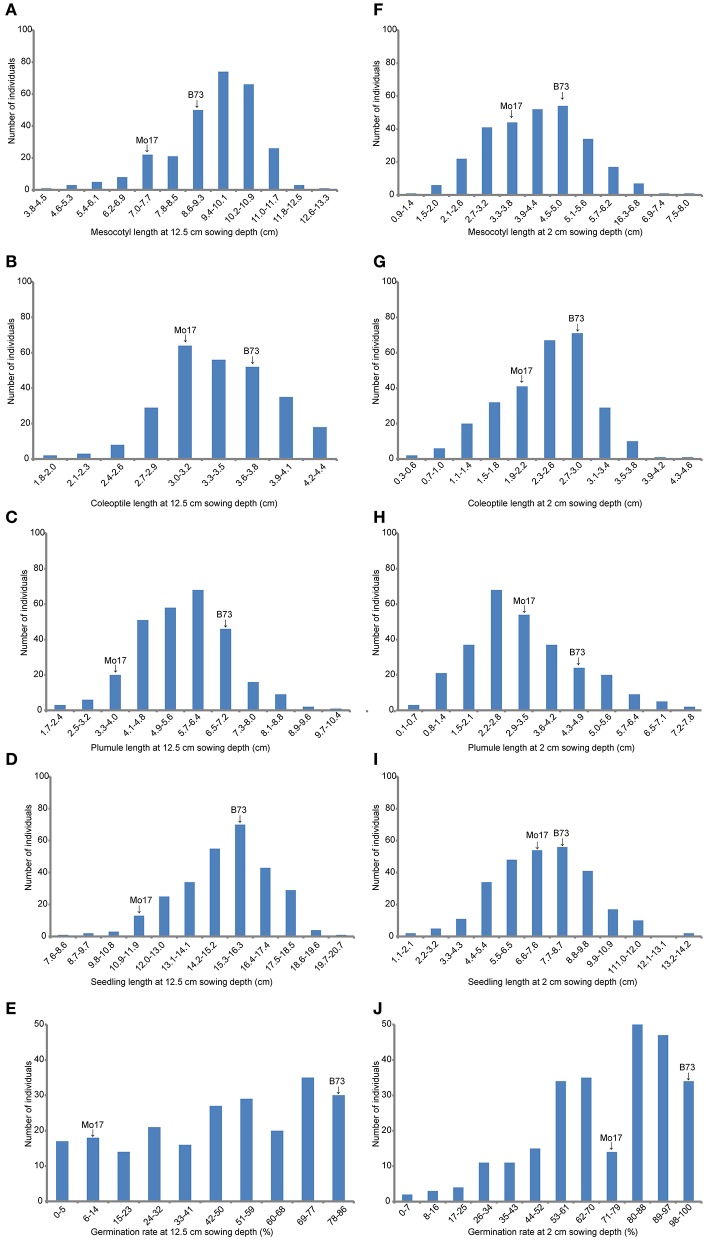
**The histogram of frequency distribution of germination-related traits under two sowing depths in IBM Syn10 population. (A)** Mesocotyl length at 12.5 cm sowing depth; **(B)** Coleoptile length at 12.5 cm sowing depth; **(C)** Plumule length at 12.5 cm sowing depth; **(D)** Seedling length at 12.5 cm sowing depth; **(E)** Germination rate at 12.5 cm sowing depth; **(F)** Mesocotyl length at 2 cm sowing depth; **(G)** Coleoptile length at 2 cm sowing depth; **(H)** Plumule length at 2 cm sowing depth; **(I)** Seedling length at 2 cm sowing depth; **(J)** Germination rate at 2 cm sowing depth.

At deep-sowing depth (12.5 cm), five germination-related traits were measured: germination rate (DSGR), mesocotyl length (DSML), coleoptile length (DSCL), plumule length (DSPL), and seedling length (DSSL). As shown in Table 1 and Figure 1a, the parental lines B73 and Mo17 were significantly different for all traits. The average values of all traits within the IBM Syn10 population were closer to those of B73 than Mo17 (Table 1). DSGR (51.9%) had the highest coefficient of variation (CV), followed by DSPL, DSCL, DSML, and the lowest was for DSSL (13.1%). DSML (79.4%) had the highest repeatability (*R*^2^), followed by DSGR, DSPL, DSSL, and the lowest was DSCL (60.3%). Skewed distributions were found in the frequency distribution histograms of DSML, DSCL, DSPL, and DSSL while irregular distribution was obtained for DSGR (Figure 2).

**Table 1:** **Germination-related traits and their heritability of Syn10 population under two sowing depths**.

**Traits^a^**	**Parents**^b^		**Syn10 population**			
	**B73**	**Mo17**	**Range**	**Mean**	**CV^c^ (%)**	***R*^2^^d^ (%)**
DSGR (%)	80.0^**^	8.9	0.0–100.0	55.8	51.9	74.1
DSML (cm)	9.0^*^	7.7	4.3–12.7	8.4	15.3	79.4
DSCL (cm)	3.7^*^	3.0	1.8–5.1	3.5	15.7	60.3
DSPL (cm)	6.8^**^	3.3	2.0–9.7	5.7	23.5	73.6
DSSL (cm)	15.8^**^	11.0	8.1–19.7	15.1	13.1	64.6
SGGR (%)	100.0^**^	78.0	6.7–100.0	73.1	29.5	81.2
SGML (cm)	4.6^**^	3.7	0.9–7.1	4.0	28.6	68.5
SGCL (cm)	3.0^*^	2.1	0.4–4.6	2.4	33.8	51.8
SGPL (cm)	4.6^*^	3.2	0.4–7.6	3.1	42.8	63.7
SGSL (cm)	8.8^*^	6.6	1.6–11.8	7.2	29.1	55.9

a *DSGR, DSML, DSCL, DSPL, DSSL represents germination rate, mesocotyl length, coleoptile length, plumule length, and seedling length under deep-sowing condition (12.5 cm), respectively; SGGR, SGML, SGCL, SGPL, SGSL represents germination rate, mesocotyl length, coleoptile length, plumule length, and seedling length under standard-germination condition (**2 cm**), respectively*.

b Means followed by the symbol ^*^ and ^**^ differ at 5% level and 1% level of significance by Student's t-test, respectively.

c *CV: coefficient of variation*.

d *R^2^: repeatability*.

Under standard-germination condition (**2 cm**), there were also significant differences between B73 and Mo17 for all traits (Table 1). However, germination rate, plumule length, and seedling length showed smaller differences than the corresponding traits under deep-sowing condition. In addition, the average values of all traits within IBM Syn10 population were closer to those of Mo17 than to B73 (Table 1). SGPL (42.8%) had the highest CV while SGML (28.6%) had the lowest one. SGGR (81.2%) had the highest *R*^2^ while that of SGCL (51.8%) was lowest. Similarly, the skewed distributions occurred in the frequency distribution histograms of SGML, SGCL, SGPL, and SGSL while the irregular distribution occured for SGGR (Figure 2).

Seedling was divided into three segments: mesocotyl, coleoptile and plumule (Figure 1b). Its length is the sum of mesocotyl and coleoptile lengths, or mesocotyl and plumule lengths, before or after plumule protrudes from coleoptile. DSSL (*p* < 0.001) and SGSL (*p* < 0.05) were significantly correlated with DSGR and SGGR, respectively (Table 2). The lengths of seedlings' three segments were also significantly correlated with their germination rates under two sowing depths except that no significant correlation existed between SGML and SGGR, or DSPL and DSML (Table 2).

**Table 2:** **Correlation coefficients (***r***) between mesocotyl length, coleoptile length, plumule length, seeding length, and germination rate of IBM Syn10 population under two sowing depths**.

**Traits**	**DSGR (%)**	**DSML (cm)**	**DSCL (cm)**	**DSPL (cm)**	**DSSL (cm)**	**SGGR (%)**	**SGML (cm)**	**SGCL (cm)**	**SGPL (cm)**	**SGSL (cm)**
DSGR (%)										
DSML (cm)	0.712^***^									
DSCL (cm)	0.165^**^	−0.341^***^								
DSPL (cm)	0.574^***^	0.018	0.693^***^							
DSSL (cm)	0.904^***^	0.740^***^	0.217^***^	0.685^***^						
SGGR (%)	0.087	0.001	0.056	0.055	0.037					
SGML (cm)	0.019	0.030	−0.003	−0.004	0.019	0.095				
SGCL (cm)	0.015	−0.032	0.031	0.043	0.007	0.158^*^	0.391^***^			
SGPL (cm)	−0.065	−0.097	0.030	0.069	−0.024	0.205^***^	0.370^***^	0.630^***^		
SGSL (cm)	−0.032	−0.047	0.018	0.044	−0.005	0.187^**^	0.793^***^	0.628^***^	0.859^***^	

*DSGR, DSML, DSCL, DSPL, DSSL represents germination rate, mesocotyl length, coleoptile length, plumule length, and seedling length under deep-sowing condition (12.5 cm), respectively; SGGR, SGML, SGCL, SGPL, SGSL represents germination rate, mesocotyl length, coleoptile length, plumule length, and seedling length under standard-germination condition (**2 cm**), respectively*.

*, **, ***, *significant correlation at p = 0.05, p = 0.01, p = 0.001, respectively*.

### QTL analysis for germination-related traits under different sowing depths

**Figure 3:**
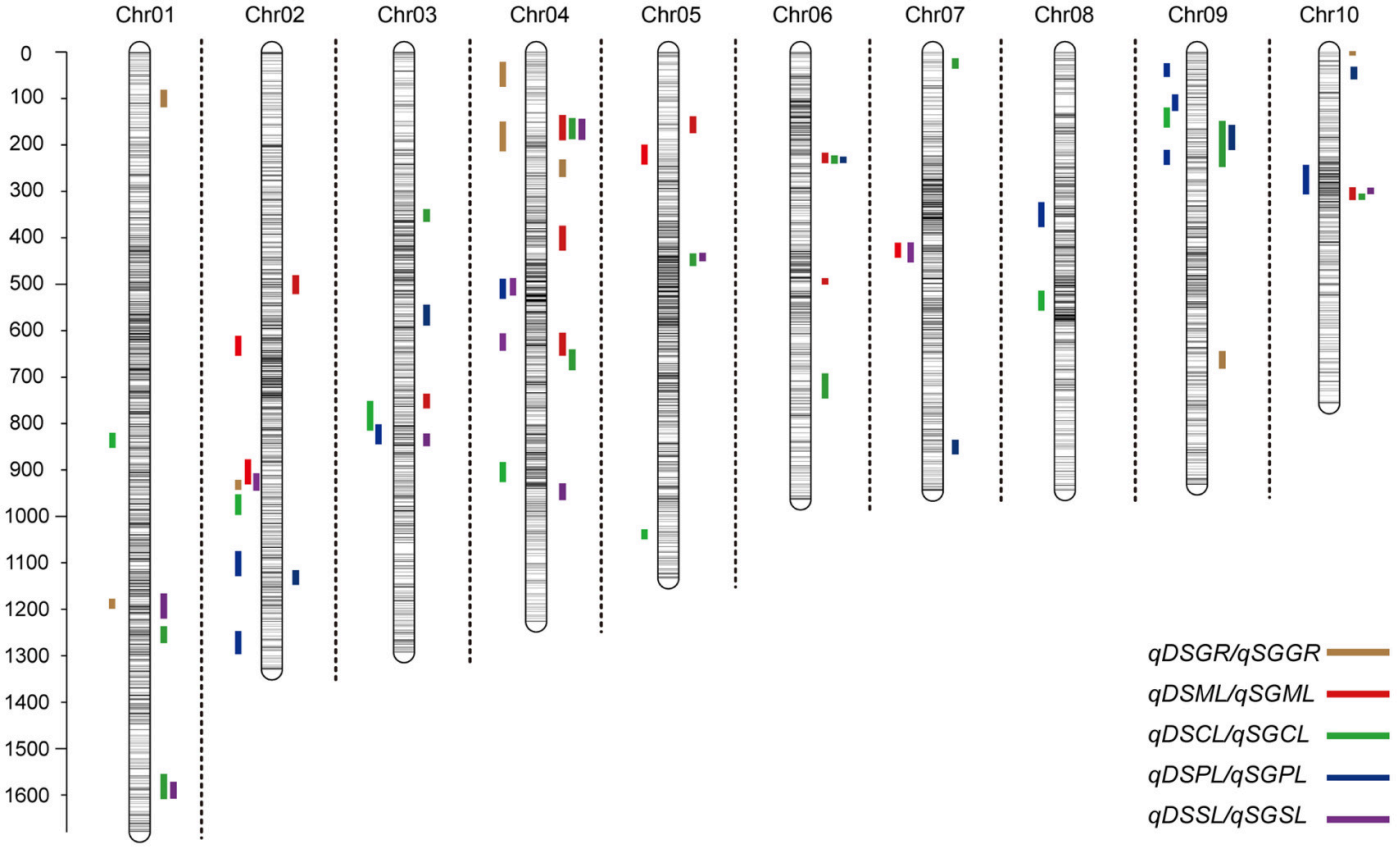
**Chromosomal location of quantitative trait loci (QTL) for deep-sowing germination ability (present in each chromosome left) and standard germination ability (present in each chromosome left) in IBM Syn10 population**. DSGR, DSML, DSCL, DSPL, DSSL represents germination rate, mesocotyl length, coleoptile length, plumule length, and seedling length under 12.5 cm sowing depth; SGGR, SGML, SGCL, SGPL, SGSL represents germination rate, mesocotyl length, coleoptile length, plumule length, and seedling length under 2 cm sowing depth, respectively.

Under deep-sowing condition (12.5 cm), LOD values for five germination-related traits varied from 2.52 to 6.65. A total of 28 QTL were detected on nine out of ten chromosomes except chromosome 6 (Table 3; Figure 3). There were four, four, seven, nine, four QTL controlling DSGR, DSML, DSCL, DSPL, DSSL, respectively. For DSGR, four QTL explained 3.19–6.33% of the total phenotypic variation. *qDSGR2-1* was located near chr02.2055.5 (205.2–205.9 Mb) on chromosome 2 and had the highest LOD values of 5.09, additive effects of –8.05 and phenotypic variation of 6.33%. For DSML, four QTL explained 3.47–4.37% of the total phenotypic variation. *qDSML5-1* was located near chr05.73.5 (6.5–7.9 Mb) and had the highest LOD values of 3.56, additive effects of –0.33 and phenotypic variation of 4.37%. Trait DSCL contained seven QTL and explained 3.21–7.27% of the total phenotypic variation. *qDSCL5-1* was also located on chromosome 5 near chr05.2131.5 (213–213.4 Mb) and had the highest LOD values of 5.98, additive effects of 0.17 and phenotypic variation of 7.27%. DSPL contained most QTL (9) and explained (2.86–7.8%) of the total phenotypic variation. *qDSPL10-1* had the highest LOD values of 6.65, additive effects of 0.4 and phenotypic variation of 7.8% and was located near chr10.1044.5 (94–111.3 Mb). Finally, four QTL in DSSL explained 3.2–4.55% of the total phenotypic variation. *qDSSL4-1* had the highest LOD values of 3.77, additive effects of –0.44 and phenotypic variation of 4.55%. *qDSSL4-1* was located near chr04.1198.5 (82.25–127.075 Mb) on chromosome 4. It was interesting to note that there were four overlapping QTL regions. *qDSGR2-1, qDSML2-2*, and *qDSSL2-1* overlapped between 205.2 and 205.6 Mb on chromosome 2. *qDSCL3-1* and *qDSPL3-1* overlapped between 184.6 and 185.25 Mb on chromosome 3. *qDSPL4-1* and *qDSSL4-1* overlap between 82.25 and 127.075 Mb on chromosome 4. *qDSML7-1* and *qDSSL7-1* overlap between 125.55 and 129.8 Mb in chromosome 7. All the values of additive effects of QTL controlling DSGR were negative, suggesting that the alleles from B73 background reduce deep-sowing germination ability.

**Table 3:** **Genome-wide QTL identification under deep-sowing condition in IBM Syn10 population**.

**QTL Symbol^a^**	**Chr**	**Position (cM)**	**Peak marker**	**LOD^b^**	***R*^2^^c^(%)**	**Additive**	**2-LOD confidence interval**			
						**effect^d^**	**PC^e^ start(Mb)**	**PC end(Mb)**	**GC^f^ start(cM)**	**GC end(cM)**
*qDSGR1-1*	1	1183.55	chr01.2532.5	2.72	3.32	−5.52	251.875	257.8	1167.45	1210.7
*qDSGR2-1*	2	932.45	chr02.2055.5	5.09	6.33	−8.05	205.2	205.9	921.95	943.9
*qDSGR4-1*	4	42.65	chr04.28.5	3.1	3.82	−6.21	2.4	3.4	29.75	72.7
*qDSGR4-2*	4	193.3	chr04.74.5	2.58	3.19	−5.45	5.875	9.7	157	212.25
*qDSML2-1*	2	646.85	chr02.1280.5	2.92	3.58	0.31	104.45	136.9	621.5	654.35
*qDSML2-2*	2	911.95	chr02.2043.5	3.55	4.34	−0.32	203.05	205.6	891.45	936.4
*qDSML5-1*	5	223.2	chr05.73.5	3.56	4.37	−0.33	6.5	7.9	201.35	235.35
*qDSML7-1*	7	423.6	chr07.1280.5	2.85	3.47	−0.29	125.55	129.8	408.5	432.5
*qDSCL1-1*	1	841.45	chr01.1955.5	3.5	4.17	0.12	194.55	195.8	828.2	849.7
*qDSCL2-1*	2	974.7	chr02.2099	2.85	3.38	0.1	207.5	210.725	958.55	992.25
*qDSCL3-1*	3	771.6	chr03.1827.5	2.54	3.21	0.1	180.95	185.25	751.95	805.25
*qDSCL4-1*	4	893.2	chr04.2021.5	3.58	4.27	0.13	200.6	216.5	883.5	913.2
*qDSCL5-1*	5	1051.15	chr05.2131.5	5.98	7.27	0.17	213	213.4	1046.85	1057.2
*qDSCL8-1*	8	525.4	chr08.1230.5	2.75	3.27	−0.1	119.675	128.65	516.45	547.25
*qDSCL9-1*	9	142.25	chr09.81.5	2.84	3.55	0.11	7.8	8.4	125.4	156.95
*qDSPL2-1*	2	1101	chr02.2192	2.74	3.29	0.25	216.7	221.6	1075.6	1122.2
*qDSPL2-2*	2	1282.4	chr02.2342.5	2.99	3.4	−0.26	232.8	234.7	1256.4	1295.6
*qDSPL3-1*	3	818.45	chr03.1873.5	6.42	7.55	0.39	184.6	188.7	802.35	829.9
*qDSPL4-1*	4	508.65	chr04.1083.5	2.52	2.86	−0.24	76.4	140.1	492.2	535.8
*qDSPL8-1*	8	345.25	chr08.244.5	2.76	3.14	0.26	22.8	53.075	327.05	370.5
*qDSPL9-1*	9	68.35	chr09.44.5	3.91	5.03	0.37	4.4	5	60.85	73.1
*qDSPL9-2*	9	98.2	chr09.68	2.58	2.92	−0.29	6.175	7.6	96.35	118.6
*qDSPL9-3*	9	221.6	chr09.116.5	4.29	4.89	0.31	10.975	12.8	206.55	230.2
*qDSPL10-1*	10	301.1	chr10.1044.5	6.65	7.8	0.4	94	111.3	297.55	309.7
*qDSSL2-1*	2	932.45	chr02.2055.5	3.21	3.87	−0.43	203.6	205.9	905.1	943.9
*qDSSL4-1*	4	510.8	chr04.1198.5	3.77	4.55	−0.44	82.25	127.075	498.25	521.9
*qDSSL4-2*	4	626.6	chr04.1623.5	2.67	3.2	0.39	158.1	165.475	609.45	636.25
*qDSSL7-1*	7	423.25	chr07.1279.5	3.23	3.88	−0.42	125.55	130.5	406	444.3

a *DSGR, DSML, DSCL, DSPL, DSSL represents germination rate, mesocotyl length, coleoptile length, plumule length, and seedling length under 12.5 cm sowing depth, respectively*.

b *LOD: Log10-likelihood value*.

c *R^2^: The coefficient of determination, which represents the percentage of phenotypic variance explained by a putative QTL*.

d *Additive effect: The positive value means that the allele from B73 is positive contributor*.

e *PC: Physical coordinates refer to B73_RefGen_v3*.

f *GC: Genetic coordinates*.

Under standard-germination condition (2 cm), the LOD values for the five germination-related traits varied from 2.52 to 7.13. A total of 37 QTL were detected on nine chromosomes, except for chromosome 8 (Table 4; Figure 3). There were four, nine, eleven, six, seven QTL controlling SGGR, SGML, SGCL, SGPL, SGSL, respectively. For SGGR, four QTL explained 2.86–4.47% of the total phenotypic variation. *qSGGR10-1* was located near chr10.0.5 (0.025–0.3 Mb) and had the highest LOD values (3.91), additive effects (–5.52), and phenotypic variation (4.47%). For SGML, nine QTL explained 2.97–5.53% of the total phenotypic variation. *qSGML6-2* was located near chr06.1288 (128.1–129.65 Mb) and had the highest LOD values (5.18), additive effects (0.34), and phenotypic variation (5.53%). For SGCL, 11 QTL explained 2.8–6.36% of the total phenotypic variation. *qSGCL7-1* was located near chr07.28.5 (2.7–2.8 Mb) and had the highest LOD values (6.06), additive effects (0.19), and phenotypic variation (6.36%). For DSPL, six QTL explained 2.51–5% of the total phenotypic variation. *qSGPL6-1* was located near chr06.932.5 (92.95–93.15 Mb) and had the highest LOD values (4.73), additive effects (–0.39), and phenotypic variation (5%). For DSSL, seven QTL explained 2.78–7.73% of the total phenotypic variation. *qSGSL10-1* was located near chr10.1096.5 (90.9–110.125 Mb) and had the highest LOD values (7.13), additive effects (0.67), and phenotypic variation (7.73%). It was also interesting to find that there were eight overlapping regions for some QTL. *qSGCL1-2* and *qSGSL1-2* overlapped between 293.5 and 295.8 Mb on chromosome 1. On chromosome 4, *qSGML4-1* overlapped *qSGCL4-1* between 5.875 and 7.2 Mb while *qSGML4-3* overlapped *qSGCL4-2* between 166.975 and 167.6 Mb. *qSGCL5-1* and *qSGSL5-1* overlapped between 39.5 and 48.05 Mb on chromosome 5. *qSGML6-1, qSGCL6-1*, and *qSGPL6-1* overlapped between 92.95 and 93.15 Mb on chromosome 6. *qSGCL9-1* and *qSGPL9-1* overlapped between 8.5 and 11.1 Mb on chromosome 9. On chromosome 10, *qSGML10-1* overlapped *qSGCL10-1* between 111.3 and 115.075 Mb, but *qSGML10-1* overlapped *qSGSL10-1* between 97.15 and 110.125 Mb.

**Table 4:** **Genome-wide QTL identification under standard-germination condition in IBM Syn10 population**.

**QTL Symbol^a^**	**Chr**	**Position (cM)**	**Peak marker**	**LOD^b^**	***R*^2^^c^ (%)**	**Additive effect^d^**	**2-LOD confidence interval**			
							**PC^e^ start (Mb)**	**PC end (Mb)**	**GC^f^ start (cM)**	**GC end (cM)**
*qSGGR1-1*	1	107.35	chr01.56.5	2.63	2.97	−4.56	5	6.4	83.6	119.15
*qSGGR4-1*	4	260.95	chr04.115.5	2.53	2.86	4.49	11.1	13.4	250.9	284.2
*qSGGR9-1*	9	680.45	chr09.1431.5	2.86	3.23	−4.99	142.7	144.2	657.95	696.25
*qSGGR10-1*	10	0.05	chr10.0.5	3.91	4.47	−5.52	0.025	0.3	0.05	7.2
*qSGML2-1*	2	501.6	chr02.351.5	3.87	4.06	0.26	30.4	39.5	479.75	520.55
*qSGML3-1*	3	754.45	chr03.1810.5	4.35	4.72	0.28	180.2	182.575	743.7	771.6
*qSGML4-1*	4	170.05	chr04.61.5	3.43	3.64	−0.25	5.2	7.8	136.8	196.5
*qSGML4-2*	4	401.85	chr04.276.5	2.85	3.1	−0.22	23.45	34.4	382.15	430.05
*qSGML4-3*	4	614.1	chr04.1595.5	2.84	2.97	0.24	157.25	167.6	602.65	654.85
*qSGML5-1*	5	159.85	chr05.54.5	4.35	4.93	0.28	4.725	5.8	138.7	169.55
*qSGML6-1*	6	221.6	chr06.925.5	3.46	3.64	−0.25	90.725	93.15	210.9	239.15
*qSGML6-2*	6	493.3	chr06.1288	5.18	5.53	0.34	128.1	129.65	487.95	499.35
*qSGML10-1*	10	304.7	chr10.1096.5	4.66	4.92	0.29	97.15	116.7	298.25	321.1
*qSGCL1-1*	1	1258.35	chr01.2654.5	2.76	2.81	−0.13	260.575	269.275	1239.05	1275.15
*qSGCL1-2*	1	1582.8	chr01.2935.5	2.85	2.91	−0.13	292.725	295.8	1558.85	1605.8
*qSGCL3-1*	3	349.1	chr03.152	5.13	5.35	0.17	14.65	17.6	341.6	362.0
*qSGCL4-1*	4	171.1	chr04.63	4.18	4.32	−0.16	5.875	7.2	152.0	190.4
*qSGCL4-2*	4	659.1	chr04.1699	3.4	3.49	0.14	166.975	172.275	641.6	687.4
*qSGCL5-1*	5	445.8	chr05.443	2.85	3.01	0.13	39.5	52.875	433.65	457.2
*qSGCL6-1*	6	229.15	chr06.932.5	3.82	3.93	−0.15	91.9	93.15	217.7	239.15
*qSGCL6-2*	6	734.55	chr06.1582.5	2.52	2.69	−0.12	155.45	159.2	697.15	756.05
*qSGCL7-1*	7	39.55	chr07.28.5	6.06	6.36	0.19	2.7	2.8	33.1	54.55
*qSGCL9-1*	9	181.15	chr09.95.5	2.65	2.8	−0.12	8.3	13.4	151.6	247.0
*qSGCL10-1*	10	312.9	chr10.1131.5	5.32	5.55	0.18	111.3	115.075	309.7	317.55
*qSGPL2-1*	2	1145.45	chr02.2239.5	3.81	3.78	0.31	221.325	225.2	1120.45	1153.3
*qSGPL3-1*	3	578.75	chr03.1607	3.23	3.29	0.27	158.5	162.1	549.7	590.9
*qSGPL6-1*	6	229.15	chr06.932.5	4.73	5	−0.39	92.95	93.15	223.4	236.65
*qSGPL7-1*	7	862.05	chr07.1728	3.41	3.37	0.27	171.525	172.875	845.6	871.45
*qSGPL9-1*	9	181.15	chr09.95.5	3.47	3.52	−0.27	8.5	11.1	164.35	210.55
*qSGPL10-1*	10	55.3	chr10.26.5	2.56	2.51	−0.25	2.4	2.6	43.45	65.3
*qSGSL1-1*	1	1188.55	chr01.2533.5	2.68	2.78	−0.38	252.95	258.05	1172.8	1220.1
*qSGSL1-2*	1	1589.65	chr01.2951.5	3.26	3.43	−0.43	293.5	295.8	1575.3	1605.8
*qSGSL3-1*	3	824.15	chr03.1884.5	3.73	4.09	0.47	187.2	191.325	816.3	841.7
*qSGSL4-1*	4	171.1	chr04.63	3.49	3.68	−0.45	5.875	7.4	154.5	193.3
*qSGSL4-2*	4	940.75	chr04.2284.5	3.20	3.37	0.46	225.25	229.4	931.1	963.65
*qSGSL5-1*	5	439.7	chr05.403.5	3.93	4.16	0.51	39.5	48.05	433.65	449.0
*qSGSL10-1*	10	304.7	chr10.1096.5	7.13	7.73	0.67	90.9	110.125	294.7	307.2

a *SGGR, SGML, SGCL, SGPL, SGSL represents germination rate, mesocotyl length, coleoptile length, plumule length and seedling length under **2 cm** sowing depth, respectively*.

b *LOD: Log10-likelihood value*.

c *R^2^: The coefficient of determination, which represents the percentage of phenotypic variance explained by a putative QTL*.

d *Additive effect: The positive value means that the allele from B73 is positive contributor*.

e *PC: Physical coordinates refer to B73_RefGen_v3*.

f *GC: Genetic coordinates*.

Combining the results under two sowing conditions, three overlapping regions were found for some QTL. Such as, on chromosome 9, *qDSCL9-1* overlapped *qSGCL9-1* between 8.3 and 8.4 Mb while *qDSPL9-3* overlapped *qSGPL9-1* between 10.975 and 11.1 Mb. In addition, overlapping of *qDSPL2-1* and *qSGPL2-1* occurred on chromosome 2 between 221.325 and 221.6 Mb. It was worth noting that all the germination-related traits under the two conditions consisted of 32 overlapping QTLs, which formed 11 QTL clusters spreading on all chromosomes except chromosome 8 (Supplementary Table 3). The result suggests that the minor effect genes play a pleiotropic role in regulating various traits.

### Real-time PCR validation

**Figure 4:**
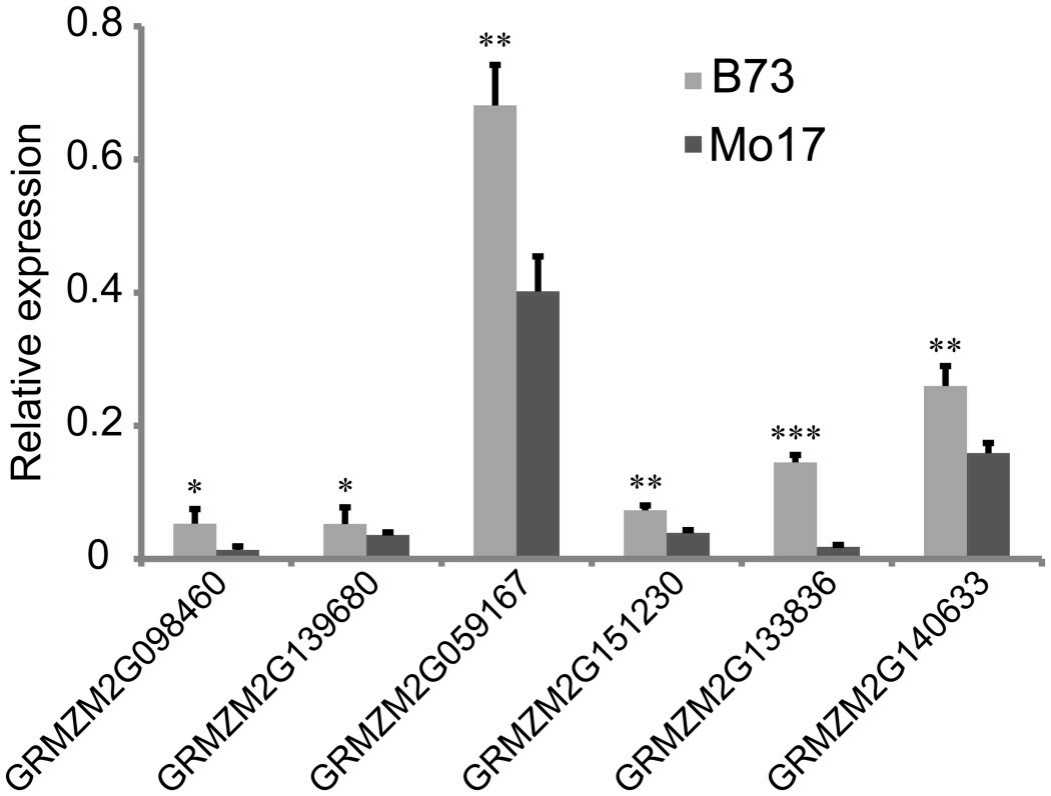
**Relative expression of six candidate genes in 7-day-old seedlings between parental lines B73 and Mo17**. Means followed by the symbol ^*, **, ***^ at 5, 1, 0.1% level of significance by Student's *t*-test, respectively.

From the overlapping intervals between different deep-sowing germination traits (Supplementary Table 1), six candidate genes related to deep-sowing germination ability were selected for expression analysis between parental lines B73 and Mo17. Among them, four candidate genes (*GRMZM2G098460, GRMZM2G139680, GRMZM2G059167, GRMZM2G151230*) were located at the overlapping region (82.25~127.075 Mb) of *qDSPL4-1* and *qDSSL4-1* in chromosome 4. The other two (*GRMZM2G133836, GRMZM2G140633*) were located at the overlapping region (125.55~129.8 Mb) of *qDSML7-1* and *qDSSL7-1* on chromosome 7. The results of Real-Time PCR experiments showed that under 12.5 cm sowing depth, all the candidate genes had significantly higher expression (*p* < 0.05) in 7-day-old seedlings of B73 than in that of Mo17 (Figure 4).

## Discussion

Maize grown in the Corn Belts of China and the United States is commonly planted 2–5 cm and ~6 cm deep, respectively. In the arid southwestern region of the United States and parts of western Mexico, Native Americans plant their local varieties of corn at a depth of 30 cm or more in an attempt to reach soil moisture (Troyer, 1997). Deep sowing, naturally allows soil moistened by the underground water to promote seed germination (Takahashi et al., 2001). However, to germinate from deep layers of soil, seed firstly has to elongate some organs such as mesocotyl. Further growth of the seedling then depends on the elongation of plumule enveloped by coleoptile. In current case, the elongations of mesocotyl, plumule, and coleoptile all contributed to deep-sowing germination. According to the correlation coefficients, the contribution to deep-sowing germination ability could be ranked as the following order: mesocotyl>plumule>coleoptile. This result was consistent with their ratios to seedling length. Under deep-sowing condition, the ratios of mesocotyl length, plumule length, and coleoptile length to seedling length were 0.625, 0.375, and 0.229, respectively. Despite this order remained unchanged for standard-germination condition, there were no significant correlations for all traits between the two sowing depths. Of course, it is crucial that mesocotyl has to be elongated because its length correlates mostly with the deep-sowing germination rate based on 280 IBM Syn10 DH lines (Table 2). This observation is consistent with previous studies (Troyer, 1997; Zhao et al., 2010), pointing mesocotyl elongation act as the single crucial response to make germplasm tolerant to deep-sowing. However, our results showed that coleoptile and plumule elongations also play important roles in the process of deep-sowing germination because they are also significantly correlated with the deep-sowing germination rate (Table 2). Recent evidences obtained in wheat study have shown that longer coleoptile is a desirable trait for sowing under drought stress environments with moisture available in the deeper layers of the soil (Singh et al., 2015).

To determine whether the genetic basis of different germination-related traits is similar or particular, we measured five germination-related traits and compared with the respective QTL. Most of the phenotypic traits in maize were controlled by small effect genes/ QTLs which individually explained only less than 3% to at most 7.8% of the phenotypic variance. Firstly, those genes or QTLs may locate in different chromosomes act as single effect gene to control the phenotype trait. As the result, this gene's effect was small to explain the phenotypic variance. But, in some other cases, genes/QTLs were clustered in the same position act as pleiotropic effects, resulting big effects to explain the phenotypic variance. This depends on the gene's function and classification, such like some resistant disease genes clustered in 3.04 and 6.02 chromosomes with huge phenotypic variance (Brefort et al., 2014; Zambrano et al., 2014). Secondly, the genetic variance for deep-sowing between Mo17 and B73 was significant, but some other factors may also affect the QTL results, such as environment and genetic × environment (G × E). Finally, we constructed a high-density genetic map with 6,618 recombination bin markers to detect the QTLs. Comparing with the traditional markers, the high density markers can help us to understand the small effect QTLs which tightly clustered in the same region, breaking the big QTLs into different pieces. The same results were also found in our previous study. Almost 91 and 92% QTLs for flowering time and plant height with small effects below 10% were found in the same population, but most of cloned genes can be found in the QTL confidence interval (Liu et al., 2015). Based on the three reasons, our results showed not big effects of phenotypic variance. In addition, under the deep-sowing and standard germination conditions, we respectively identified five and seven overlapping regions for different traits (Figure 3). For example, *qDSGR2-1, qDSML2-2*, and *qDSSL2-1* overlapped on chromosome 2 between 205.2 and 205.6 Mb while *qSGML6-1, qSGCL6-1*, and *qSGPL6-1* overlapped on chromosome 6 between 92.95 and 93.15 Mb. Significant correlations existed between any two traits of the corresponding three traits: germination rate, mesocotyl length, seedling length under 12.5 cm sowing depth or mesocotyl length, coleoptile length, plumule length under 2 cm sowing depth (Table 2). Furthermore, total 11 clusters were found across both growing conditions and overlapping QTL loci were consistent with significant correlations between different germination-related traits (Supplementary Table 3). Similar results were obtained in previous studies (Tommasini et al., 2008; Zhao et al., 2010; Zhang et al., 2012).

For sowing depth at 25 cm, Troyer (1997) evaluated 26 translocation stocks and found that the dominant genes responsible for mesocotyl length were located on the short arms of chromosome 3 and 9 as well as near the centromere of chromosome 6. Under our deep-sowing condition (at 12.5 cm), no QTL for mesocotyl length was found on above corresponding regions. Instead, four QTL controlling coleoptile length (*qDSCL9-1*) or plumule length (*qDSPL9-1, qDSPL9-2, qDSPL9-3*) were located on the short arm of chromosome 9. In contrast, one QTL for mesocotyl length (*qSGML6-1*) was found to locate near the centromere of chromosome 6 under the standard-germination condition. Recent studies at two sowing depths of 10 and 20 cm revealed 25 QTL for four traits including mesocotyl length, coleoptile length, seedling length, and germination rate in two parents of X178 and 3681-4 (Zhang et al., 2012). However, no QTL was found on chromosome 5 or 9, contrary to our findings. The difference might be related to the different sowing depths, mapping populations, as well-molecular markers (their density, coverage of certain intervals for SSR and SNP). Furthermore, we found 11 overlapping QTL regions for early seed vigor by comparing with previous data (Han et al., 2014; Trachsel et al., 2016). The results showed that there may exist more candidate genes for traits related to seed germination on chromosomes 4 and 7 (Supplementary Figure 1). In addition, to some extent, maize yield is determined by the number of harvested kernels and their individual kernel weight. According to the study of Prado et al. (2013) using IBM Syn4 population, there are overlapped QTL from our research with their results for kernel weight. The most interesting was the consistent QTL (such as *qSGML6-2*) for mesocotyl length under 2 cm sowing depth on chromosome 6 which was in agreement with QTL (between bins 6.02 and 6.05) for kernel weight reported by Prado et al. (2013).

The high resolution linkage map of IBM Syn 10 not only contributes to QTL mapping of deep-sowing germination ability and understanding of its molecular basis, but is also helpful for breeding of deep-sowing tolerant varieties via marker-assisted selection (MAS). In wheat, two QTL *qCL.4B.1* and *qCL.3B.1* emerge to be important for coleoptile length in the WL711/C306 RIL population, suggesting their potential value for use in MAS after validation for longer coleoptile length and improved establishment. Our study identified five overlapping regions (221.325~221.6 Mb on chromosome 2, 184.6~185.25 Mb on chromosome 3, 82.25~127.075 Mb on chromosome 4, 125.55~129.8 Mb on chromosome 7, 10.975~11.1 Mb on chromosome 9) related to deep-sowing germination traits. The corresponding markers closely linked with the five regions can be developed to predict deep-sowing tolerance during germination in further MAS programs.

Analysis of 107 genes that have explicit annotations led to assignment of their locations: *qDSPL2-1* and *qSGPL2-1* overlapping region between 221.325 and 221.6 Mb on chromosome 2, *qDSPL9-3* and *qSGPL9-1* overlapping region between 10.975 and 11.1 Mb on chromosome 9, *qDSCL-3* and *qDSPL-3* overlapping region between 184.6 and 185.25 Mb on chromosome 3, *qDSPL4-1* and *qDSSL4-1* overlapping region between 82.25 and 127.075 Mb on chromosome 4, *qDSML7-1* and *qDSSL7-1* overlapping region between 125.55 and 129.8 Mb on chromosome 7. These genes include hormone related proteins (gibberellin receptor GID1L2, auxin-independent growth promoter, auxin-responsive SAUR family member), transcription factors (MYB, Zinc finger protein, NAC, WRKY, bZIP, MADS, SAPK, AP2), stress response enzymes (peroxidase, cytochrome c oxidase, mitogen-activated protein kinase, glucose-6-phosphate isomerase, glutathione transferase, NADP-dependent glyceraldehyde-3-phosphate dehydrogenase, ATP synthase, glucan endo-1,3-beta-glucosidase, mitochondrial NADH ubiquinone oxidoreductase), drought-induced proteins, oxygen evolving enhancer proteins, as well as cell number regulators. Based on our previous studies (Zhao et al., 2010), some key genes such as GA receptor, MYB transcriptional factor, antioxidant enzyme, and cell growth regulator induced by deep-sowing and gibberellic acid might play a critical role in seed germination and mesocotyl elongation of deep-sowing tolerant maize inbred line 3681-4 from 20 cm deep layer of soil. For the six candidate genes that are presumably related to deep-sowing germination ability, we performed Real-Time PCR analysis. The results showed that they all had higher expression in B73 than in Mo17 under deep-sowing condition (Figure 4). These genes were related to gibberellin receptor, MYB transcription factor, oxidase enzyme, cell metabolism (such as cell number regulator and cell cycle regulator, cell wall organization, or biogenesis). According to our previous study (Zhao et al., 2010), their expression were up-regulated by deep-sowing and gibberellic acid treatment. So, our current result likely suggests that B73 has stronger deep-sowing germination ability than Mo17 but remains to be validated.

The gene *GRMZM2G133836* encodes the gibberellin receptor gene family *GID1* (*GIBBERELLIN INSENSITIVE DWARF1*). It is well-known that gibberellin signaling pathways control coat-dormancy release, endosperm weakening, and organ expansion during seed germination (Voegele et al., 2011). In our current study, *GID1* gene expression was higher in 7-day-old seedling of deep-sowing tolerant maize line B73 at 12.5 cm sowing depth. Similarly, in our previous report (Zhao et al., 2010), GA receptor *GID1* was up-regulated in 10-day-old seedling of deep-sowing tolerant maize line 3681-4 at 20 cm sowing depth when endogenous GA_3_ was applied. According to the report of Voegele et al. (2011), *GID1* played distinct roles during seed germination of *Lepidium sativum* and *Arabidopsis thaliana*. It is speculated that deep-sowing promotes both the synthesis of GA and GA receptor GID1, resulting in degradation of DELLA protein and derepression of GA inducible genes.

Gene *GRMZM2G059167*, named as MYBR38, encodes a putative MYB DNA-binding domain superfamily protein (http://www.maizegdb.org/gene_center/gene/GRMZM2G059167). Expression profile analysis of B73 indicated most expression of this gene occurred in these tissues such as shoot apical meristem, mesocotyl, stem internode, coleoptile, primary root, leaf (Sekhon et al., 2011). In our previous report (Zhao et al., 2010), MYB family members such as MYB18 and MYBAS1 were identified to be the main transcription factors that were up-regulated in 10-day-old seedling after the treatment of deep-sowing and exogenous GA_3_, which is similar to our present result. In *Hordeum vulgare*, HvGAMYB, a MYB transcription factor was shown to be expressed in barley aleurone cells in response to gibberellin during seed germination (Diaz et al., 2002). These findings confirm that MYB genes should be involved in the regulation of germination process. However, it is unclear how MYB transcription factors participate in seed germination from deep layer of soil.

Gene *GRMZM2G139680* encodes 2-cys peroxiredoxin BAS1, which belongs to peroxiredoxin (PRX) family. PRX are thiol-dependent antioxidants containing one (1-cysteine [-Cys]) or 2-Cys conserved Cys residues that protect lipids, enzymes, and DNA against reactive oxygen species (Haslekas et al., 2003). In *Brassica napus*, the PRX antioxidant proteins might play a role in protecting seed from oxidative damages and elevated calmodulin levels was indicative of increased enzymatic activities required for germination (Gao et al., 2002). Haslekas et al. (2003) found that there was no correlation between PRX levels and the duration of the after-ripening period required before germination using transgenic Arabidopsis lines overexpressing the homologous gene in barley and PRX were unlikely to contribute to maintenance of dormancy. However, over-expressed seeds were less inclined to germinate than wild-type seeds in the presence of NaCl, mannitol, and methyl viologen, suggesting that PRX can sense harsh environmental surroundings and play a part in the inhibition of germination under unfavorable conditions (Haslekas et al., 2003). It is proposed that *GRMZM2G139680* may be involved in protecting the embryo and aleurone layer surviving desiccation against damage caused by reactive oxygen species and promoting seed germination of maize when subjected to deep-sowing stress.

In our study, three candidate genes, which were associated with cell metabolism, may participate in the regulation of new cell biogenesis and embryo germination and growth. Gene *GRMZM2G151230* encodes cell number regulator 2 (*CNR2*), regulating cell numbers. Their roles in controlling fruit and organ size have been demonstrated by *FW2.2* in *Lycopersicon esculentum* (Frary et al., 2000), *ZmCNR1* and *ZmCNR2* in *Zea mays* (Guo et al., 2010), and *PavCNR12* in *Prunus avium* (De Franceschi et al., 2013). Gene *GRMZM2G140633* is expressed in mesocotyl, coleoptile and stem internode, and encodes cyclin delta-2, a member of cell cycle regulators that regulate cyclin dependent kinases (CDKs) (Sekhon et al., 2011). In *Zea mays*, Cyclin D-CDKs were active and played an important role in controlling the process of seed germination (Godinez et al., 2012). *GRMZM2G098460*, one of the six genes, is expressed in embryo, endosperm, primary root, juvenile vascular leaf, leaf base, leaf tip, and may be involved in the regulation of cell wall organization or biogenesis (Sekhon et al., 2011).

Combined with all the above analysis, we proposed the hypothesis of seed germination under deep-sowing condition. Firstly, deep-sowing promotes the synthesis of GA receptor *GID1*, resulting in the trigger of GA signaling pathway and the derepression of GA inducible genes. Then, transcription factors such as MYB, antioxidant enzymes such as PRX are activated. Finally, genes involved in cell metabolism (such as *CNR2, cyclin delta-2* and so on) are expressed, which has contributed to new cell biogenesis and embryo germination and growth. In general, it seems that the six candidate genes can be used as an excellent source of candidate gene for investigating the genetic control of seed germination and seedling growth of maize under deep-sowing condition. In our further study, we will focus on functional verification and signal transduction pathways of the six candidate genes. The results of our study represent a valuable resource for gene discovery and functional characterization of seed germination tolerant to deep-sowing in maize.

## Conclusions

Thirty-seven and twenty-eight QTL regulating maize germination ability traits under two sowing conditions (2 and 12.5 cm) were mapped in maize IBM Syn10 population. In total, 32 overlapping QTL formed 11 QTL clusters. Furthermore, we identified six candidate genes related to deep-sowing germination ability which co-located in the cluster regions. These findings will provide the genetic basis for molecular marker assisted breeding and functional studies of seed germination tolerant to deep-sowing.

## Author contributions

HL, TL, JW, and GZ designed and supervised the study. LZ, JW, CL, XZ, SX, YZ, SL, SH, and ML performed the experiments. HL, CL, and GZ analyzed the data. HL, TL, and GZ prepared the manuscript and all authors read, and approved the manuscript.

## Funding

This research was supported by National Natural Science Foundation of China (31371712, 31301290), Special Fund for Agro-scientific Research in the Public Interest of China (201303002), Zhejiang Key Project for New Variety Breeding of Agriculture (Grain) (2016C02050-9-5), Crop Germplasm Resources Protection Project of Zhejiang (2015004), Zhejiang Natural Science Foundation (LY13C130011), and Talent Project Funded by Shandong Agricultural University (72127). Authors would also like to thank USDA's National Institute of Food and Agriculture (Project Numbers: IOW04314, IOW01018), as well as the RF Baker Center for Plant Breeding and K.J. Frey Chair in Agronomy at Iowa State University for funding this work.

### Conflict of interest statement

The authors declare that the research was conducted in the absence of any commercial or financial relationships that could be construed as a potential conflict of interest.

## Supplementary material

Supplementary Figure 1 **Overlap QTL regions by comparing with previous study**. The star with yellow color represents the mQTLs for seed vigor-related traits from Han et al. (2014). DSGR, DSML, DSCL, DSPL, and DSSL represents germination rate, mesocotyl length, coleoptile length, plumule length, and seedling length under 12.5 cm sowing depth on each chromosome left, respectively; SGGR, SGML, SGCL, SGPL, SGSL represents germination rate, mesocotyl length, coleoptile length, plumule length, and seedling length under **2 cm** sowing depth on each chromosome right, respectively.

Supplementary Table 1 **A list of genes that have annotations extracted from five overlapping intervals**.

Supplementary Table 2 **The primers and T_m_, T_a_, T_e_ of six candidate genes and actin genes for Real-Time PCR**.

Supplementary Table 3 **Twenty two overlapping QTL and their clusters for different germination-related traits in Tables 3, 4**.
