# Supplementary figures and images for: Quantitative Trait Locus Analysis for Deep-Sowing Germination Ability in the Maize IBM Syn10 DH Population
Source: Front Plant Sci. 2017 May 22;8:813. doi: 10.3389/fpls.2017.00813 (PMC5439002; doi:10.3389/fpls.2017.00813)

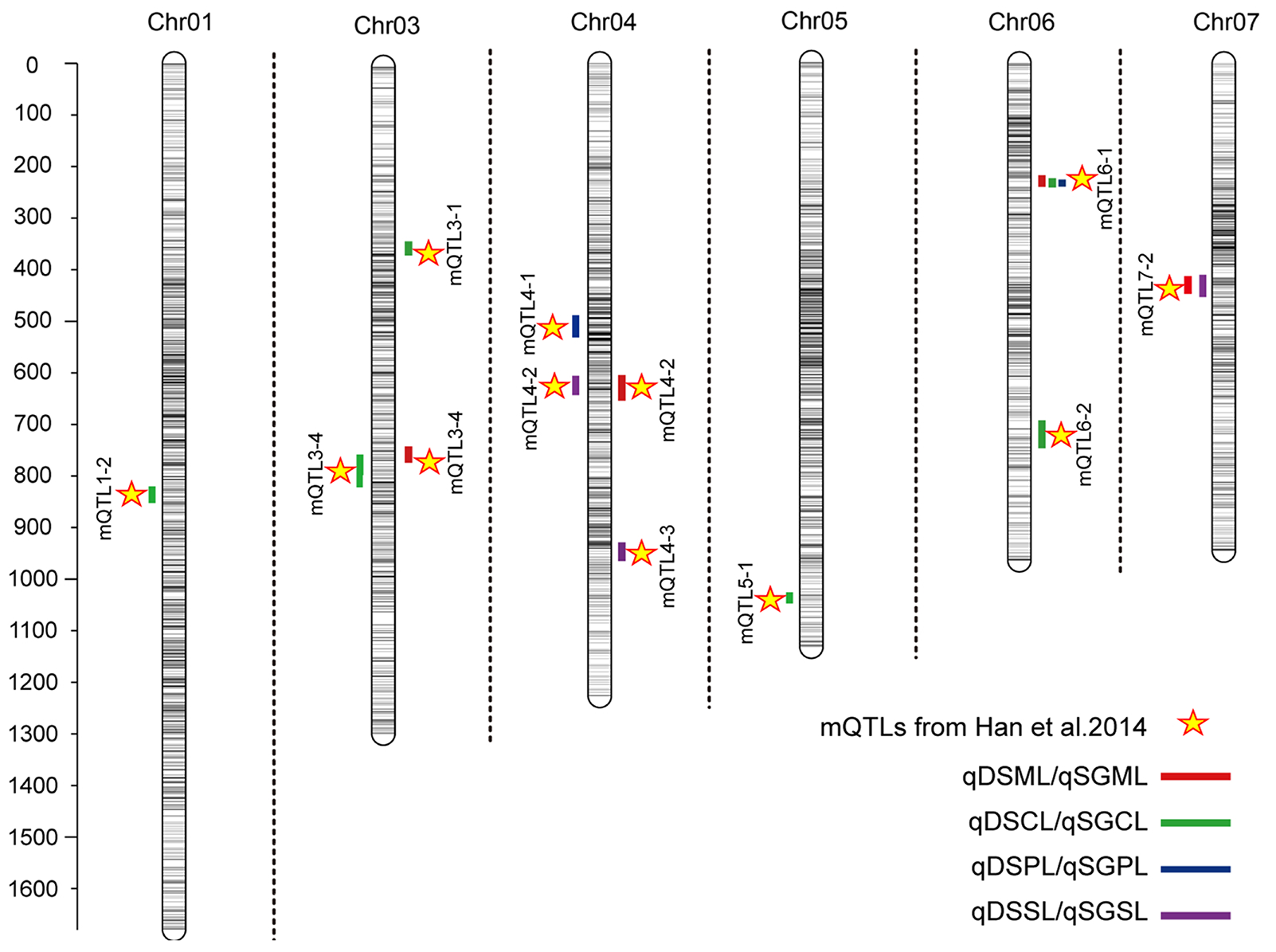

Supplement: Supplementary Figure 1 — Overlap QTL regions by comparing with previous study. The star with yellow color represents the mQTLs for seed vigor-related traits from Han et al. (2014). DSGR, DSML, DSCL, DSPL, and DSSL represents germination rate, mesocotyl length, coleoptile length, plumule length, and seedling length under 12.5 cm sowing depth on each chromosome left, respectively; SGGR, SGML, SGCL, SGPL, SGSL represents germination rate, mesocotyl length, coleoptile length, plumule length, and seedling length under 2 cm sowing depth on each chromosome right, respectively. [file Image1.TIF]
